# The Genome of *Arsenophonus* sp. and Its Potential Contribution in the Corn Planthopper, *Peregrinus maidis*

**DOI:** 10.3390/insects15020113

**Published:** 2024-02-05

**Authors:** Yu-Hui Wang, Aram Mikaelyan, Brad S. Coates, Marcé Lorenzen

**Affiliations:** 1 Department of Entomology and Plant Pathology, North Carolina State University, Raleigh, NC 27695, USA; ywang259@ncsu.edu (Y.-H.W.); amikael@ncsu.edu (A.M.); 2 USDA-ARS, Corn Insects & Crop Genetics Research Unit, Ames, IA 50011, USA; brad.coates@ars.usda.gov

**Keywords:** genome assembly, *Arsenophonus* sp., *Peregrinus maidis*, hemiptera

## Abstract

**Simple Summary:**

Microbes play important roles in the biochemistry, physiology and survival of insects. An example is *Arsenophonus* sp. in the brown planthopper (*Nilaparvata lugens*), an endosymbiotic bacterium supplementing the nutritionally poor diet of its sap-feeding host. In this study, we use the genome of *Nl Arsenophonus* sp. as a reference to facilitate the genome assembly of a closely related *Arsenophonus* sp. found in the corn planthopper (*Peregrinus maidis*). Our assembly is one of the largest *Arsenophonus* genomes reported to date. We also investigated the role of *Arsenophonus* sp. in *P. maidis*, in which it appears to provide B vitamins and essential amino acids.

**Abstract:**

The co-evolution between symbionts and their insect hosts has led to intricate functional interdependencies. Advances in DNA-sequencing technologies have not only reduced the cost of sequencing but, with the advent of highly accurate long-read methods, have also enabled facile genome assembly even using mixed genomic input, thereby allowing us to more easily assess the contribution of symbionts to their insect hosts. In this study, genomic data recently generated from *Peregrinus maidis* was used to assemble the genome of a bacterial symbiont, *Pm Arsenophonus* sp. This ~4.9-Mb assembly is one of the largest *Arsenophonus* genomes reported to date. The Benchmarking Universal Single-Copy Orthologs (BUSCO) result indicates that this *Pm Arsenophonus* assembly has a high degree of completeness, with 96% of the single-copy Enterobacterales orthologs found. The identity of the *Pm Arsenophonus* sp. was further confirmed by phylogenetic analysis. Kyoto Encyclopedia of Genes and Genomes (KEGG) pathway analysis indicates a major contribution by *Pm Arsenophonus* sp. to the biosynthesis of B vitamins and essential amino acids in *P. maidis*, where threonine and lysine production is carried out solely by *Pm Arsenophonus* sp. This study not only provides deeper insights into the evolutionary relationships between symbionts and their insect hosts, but also adds to our understanding of insect biology, potentially guiding the development of novel pest control methods.

## 1. Introduction

The corn planthopper, *Peregrinus maidis*, is a notorious sap-feeding pest that targets corn and sorghum, and is known to vector the maize mosaic rhabdovirus and maize stripe tenuivirus [1]. The combination of insect damage and viral infection can significantly reduce crop yield [2]. Current management tactics mainly rely on insecticide applications, but effective control remains difficult because of the limited direct exposure of *P. maidis* under field conditions. Insecticides also have negative effects on ecosystems and human health. As such, new efficacious and sustainable methods are needed for the management of *P. maidis* and the viruses it transmits. Sequence-specificity-based RNA interference (RNAi) holds promise for the targeted control of pest species [3]. The efficacy of RNAi on *P. maidis* has been validated under laboratory conditions [4,5,6], but an efficient system for delivering double-stranded RNA (dsRNA) to *P. maidis* in the field is not yet available. Innovative solutions for delivery involve the modification of symbionts to express host-specific dsRNAs [7,8,9], but research into the symbionts of *P. maidis* is still limited. Therefore, we leveraged the genomic sequence data recently generated for *P. maidis* in order to assemble the genome of the *Arsenophonus* sp. associated with this hemipteran. We focused on *Arsenophonus* sp. because of its demonstrated nutritional importance in another planthopper, *Nilaparvata lugens* [10], which suggests it may play a similar role in *P. maidis*. Moreover, the fact that protocols have been worked out for culturing and genetically modifying other *Arsenophonus* spp. [11,12] bodes well for the development of similar methods for *Pm Arsenophonus* sp. and supports the potential of using this symbiont to deliver dsRNA to *P. maidis*.

The genus *Arsenophonus*, first proposed in 1991 for *Arsenophonus nasoniae* [13], derives its name from the Greek “Arsen” (male) and “phonus” (slayer), reflecting the ability of *A. nasoniae* to kill the male offspring of *Nasonia vitripennis*. *Arsenophonus* spp. have been found in many insect species with diverse lifestyles [14]. They serve as primary symbionts in *Lipoptena cervi* and *Aleurodicus disperses* [15,16], and as secondary symbionts in *Aleurodicus floccissimus*, *Trialeurodes vaporariorum*, *N. lugens* and triatomine bugs [10,16,17]. Such a stable association is not observed in the honeybee, *Apis mellifera*, as individuals can acquire *Arsenophonus* sp. from the environment or nestmates [18]. Although it is rare, *Arsenophonus* spp. can act as insect-vectored plant pathogens; for example, *A. phytopathogenicus* transmitted by *Pentastiridius leporinus* causes low sugar content in sugar beets [19].

In this study, we identified *Arsenophonus*-specific scaffolds within a newly generated *P. maidis* genome assembly. The ~4.9 Mb *Pm Arsenophonus* genome assembly was surveyed for genes involved in key biosynthetic pathways, and the role *Pm Arsenophonus* sp. plays in the production of B vitamins and essential amino acids was determined. The data presented here have the potential to assist with the development of novel pest control methods, both by increasing our understanding of *P. maidis* biology, and by providing a candidate for use in symbiont-mediated RNAi.

## 2. Materials and Methods

### 2.1. Insect Rearing

Corn planthoppers were reared on 5-week-old corn (cultivar Early Sunglow, Park Seed Company, Greenwood, SC, USA) and kept in 30 cm × 30 cm × 60 cm cages covered by nylon-mesh screens (BioQuip Products Inc., Compton, CA, USA) in a room at 25 ± 1 °C, ~70% RH and at a 14:10 (light:dark) photoperiod.

### 2.2. Genome Sequencing, Assembly and Annotation

We extracted genomic DNA from *P. maidis* using the Mag-Bind Blood and Tissue DNA HDQ 96 Kit (Omega Bio-tek, Norcross, GA, USA). The quality of genomic DNA was assessed and submitted to Eremid Genomic Services (North Carolina Research Campus, Kannapolis, NC, USA) for library preparation using SMRTbell Express Template Prep Kit 2.0 (Pacific Biosystems, Menlo Park, CA, USA) and subsequent high-fidelity (HiFi) read generation on a Sequel IIe system (Pacific Biosystems). Raw reads were converted to fastq format using BamTools v. 2.5.1 [20], filtered to remove reads < 8 kb, and the data was transferred to the USDA–ARS SCINet high-performance computing cluster, Ceres. A histogram of canonical k-mer counts was made at k  =  21 for filtered reads using Meryl [21], and was then applied as the maximum depth input for the R script, GenomeScope 2.0 [22,23]. Next, reads were assembled into contigs using hifiasm [24] with default assembly options but purging adjusted to [-I 3 (aggressive purging); -purge-max 14; -s 0.55; n-hap 2 (diploid)] on 40 cores with 768 GB DDR3 ECC RAM. Then, contigs were scaffolded using RagTag [25] on Galaxy Europe (https://usegalaxy.eu, accessed on 25 May 2022) [26]. RagTag uses whole-genome homology to scaffold, in which a reference genome is used to inform the order and orientation of query sequences. The genome of *Arsenophonus* sp. in *N. lugens* (accession number: GCA_000757905.1) was used as a reference to scaffold contigs [10]. Completeness of the Pm Arsenophonus assembly was compared to other Arsenophonus genome assemblies (accession number: GCA_004768525.1, GCA_020268605.1, GCA_001534665.1, GCA_900343015.1, GCA_900343025.1, GCA_002287155.1 and GCA_000757905.1) using Benchmarking Universal Single-Copy Orthologs (BUSCO) [27,28,29] on Galaxy Europe (https://usegalaxy.eu, accessed on 22 June 2022) [26]. Genes were then predicted from the genome assemblies above using Glimmer [30], and annotation of the resulting genes was conducted using the BLASTx algorithm against the non-redundant protein database (E-value cutoff of 10^−5^) through OmicsBox software (BioBam, Valencia, Spain) [31]. Then, criteria based on a previous study (E-value < 10^−15^ and similarity < 80%) [32] were applied to the predicted genes to roughly estimate the number of pseudogenes, and free-living bacteria *Proteus mirabilis*, *Escherichia coli* and *Pseudomonas aeruginosa* were included for comparison. Pathway analysis was then conducted on the genes predicted from the *Pm Arsenophonus* assembly and *P. maidis* transcriptome data [5] using the Kyoto Encyclopedia of Genes and Genomes (KEGG) [33] through OmicsBox.

### 2.3. RNA Polymerase β Subunit Gene (rpoB) Identification and Phylogenetic Analysis

The amino-acid sequence of rpoB (accession number: WP_000263098.1) was used as a query to search for rpoB orthologs in the predicted Arsenophonus genes exported from OmicsBox. The rpoB nucleotide sequences were then aligned using the MUSCLE algorithm [34] and evaluated against 24 nucleotide substitution models to find the most appropriate one for phylogenetic analysis in MEGAX [35]. The phylogenetic tree based on rpoB was constructed using the Maximum Likelihood method, with the best-fitting substitution model identified by the lowest Bayesian information criterion score. Tree topology was evaluated using bootstrap analysis with 1000 replications. The rpoB genes of *Buchnera aphidicola* and *Riesia pediculicola* were used as outgroups to root the phylogenetic tree. The predicted genes of *B. aphidicola* and *R. pediculicola* were generated from genome assemblies *B. aphidicola* JF99 (accession number: GCA_000183285.1) and *R. pediculicola* (accession number: GCA_014879315.1), and were queried for rpoB orthologs as described above.

## 3. Results and Discussion

**Figure 1:**
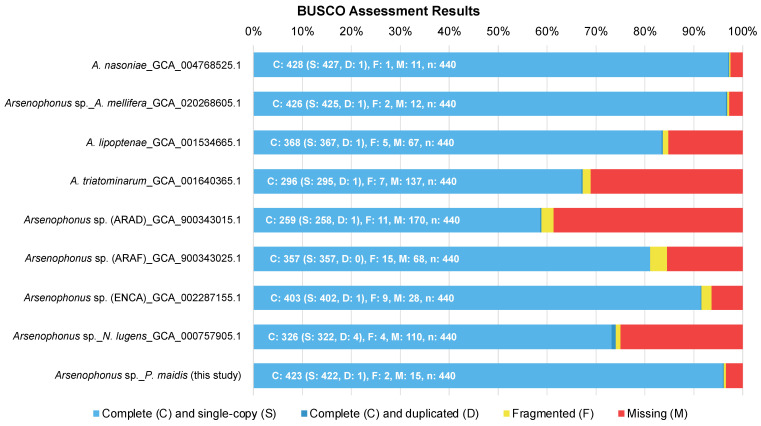
BUSCO assessment results. The completeness of genome assemblies was assessed using BUSCO on Galaxy Europe (https://usegalaxy.eu, accessed on 22 June 2022).

Eleven *Arsenophonus*-specific scaffolds were identified within the *P. maidis* genomic data. Together these formed a genome assembly of ~4.9 Mb, which was then compared to data from *Arsenophonus* spp. whose genome assemblies and roles in their respective hosts were available. An exception was made for *Arsenophonus* sp. in the keeled treehopper (*Entylia carinata*), due to the closer relationship between its insect host and *P. maidis*. Our *Pm Arsenophonus* assembly is ~4.9 Mb in size, one of the largest reported to date. While the difference in genome size might be attributed to genuine biological variation among the genomes, it could also result from the different sequencing platforms. Notably, the larger *Arsenophonus* genome assemblies have been mostly generated using third-generation PacBio and/or Nanopore reads, which generate mean read lengths that are sufficient to span repetitive regions and facilitate more accurate and complete genome assemblies. In contrast, assemblies from short-read data often leave repetitive regions unassembled, resulting in gapped pseudomolecules [36]. Therefore, the comparatively larger-sized *Pm Arsenophonus* genome could be a consequence of our use of PacBio HiFi reads. *De novo* assembly of other *Arsenophonus* genomes with long-read data may alter the ranking based on genome size. The large size of the *Pm Arsenophonus* genome may contribute to it containing 423 of the 440 single-copy orthologs in the BUSCO Enterobacterales data set (Figure 1), which is among the highest compared to other *Arsenophonus* genomes. BUSCO results also suggest that some *Arsenophonus* genome assemblies may be incomplete and thus have underestimated sizes, particularly those that are not primary symbionts (*A. triatominarum*, *Arsenophonus* sp. in *A. floccissimus* and *Arsenophonus* sp. in *N. lugens*).

During symbiont evolution, characteristic changes manifest within the genomes of host-dependent bacteria, reflecting the adaptation to different symbiotic lifestyles and ongoing selection pressures. One commonly observed trend is genome size reduction, which leads to more host-restricted symbionts [37] and the accumulation of mutations. A prevalent mutational bias towards AT suggests that primary symbionts generally tend to have genomes with a lower GC content. This is reflected in our comparative genomic analysis (Table 1). Specifically, the primary symbionts *A. lipopteni* and *Arsenophonus* sp. in *A. disperses* (hereafter “*Arsenophonus* sp. (ARAD)”) have a lower GC content, with an estimated 29.4 and 32.2%, respectively. The high 41.3% GC content of the *Pm Arsenophonus* genome and the comparatively large genome size suggest that *Pm Arsenophonus* sp. is at a relatively early stage of symbiont evolution.

Pseudogenes, or putative non-functional remnants of once-active genes, serve as another indicator of evolutionary stage and degree of endosymbiotic specialization. In the process of the transition from a free-living bacterium to a primary symbiont, pseudogenes accumulate due to the changing selective pressures associated with the symbiotic lifestyle. In a free-living state, gene function is maintained to support the diverse requirements of independent life. As an organism enters into a symbiotic relationship, a subset of genes that were essential in a free-living state might become redundant, leading to the formation of pseudogenes. Once the symbiotic relationship is well established and the organism has become a primary symbiont, there could be a deletional bias whereby non-functional DNA (like pseudogenes) is removed from the genome [37,38]. This pattern may lead to relatively higher numbers of pseudogenes in organisms that are neither fully free-living nor fully adapted as primary symbionts. Based on the criteria applied, 25 pseudogenes were estimated to be present in the genomes of *P. mirabilis*, one in *E. coli* and none in *P. aeruginosa*. Among *Arsenophonus* spp., the fewest number of pseudogenes was estimated to be present in the genomes of primary symbionts *A. lipopteni* and *Arsenophonus* sp. (ARAD) (Table 1). This was in line with expectations for free-living bacteria and primary symbionts. Interestingly, the *Pm Arsenophonus* genome appears to contain 1123 pseudogenes, which indicates that it is neither a free-living bacterium nor a primary symbiont. This fits well with the idea that *Pm Arsenophonus* sp. is at a relatively early stage of symbiont evolution.

**Figure 2:**
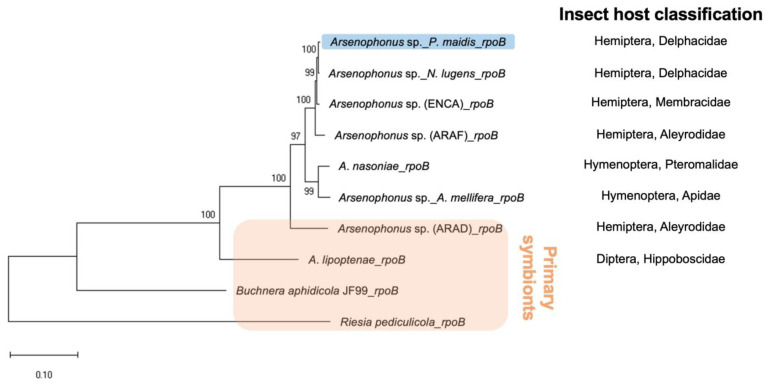
Maximum likelihood-based phylogenetic tree of *Arsenophonus* spp. estimated from a consensus *rpoB* nucleotide sequence alignment. The proportion (%) of 1000 bootstrap replicates supporting each node is indicated. The *rpoB* gene of *Pm Arsenophonus* sp. is highlighted in blue, while those identified in primary symbionts are highlighted in orange. The scale bar represents an evolutionary distance of 0.10 nucleotide substitutions per site.

The identity of *Pm Arsenophonus* sp. was further confirmed by phylogenetic analysis. The 16S ribosomal RNA (16S rRNA) gene, which produces the RNA component of the prokaryotic small ribosomal subunit, is commonly used for bacterial identification. The essential role of 16S rRNA results in its ubiquitous presence in almost all bacteria, making it a good molecular marker. Additionally, since the function of 16S rRNA has not changed, random changes in the sequence can be used to measure evolutionary time. Available 16S rRNA gene databases, such as Greengenes [39], Ribosomal Database Project [40] and SILVA [41], reflect the common use of the 16S rRNA gene. Despite the ease of using the 16S rRNA gene for bacterial identification, problems can arise [42]. For example, some species possess multiple copies of the 16S rRNA gene, and the sequences can differ by as much as 11.6% [43], which can cause identification issues. Since *Arsenophonus* spp. also possess multiple copies of the 16S rRNA gene, we used the single-copy gene, encoding the β subunit of RNA polymerase (*rpoB*) instead for phylogenetic analysis and the identification of *Arsenophonus* spp. (Figure 2). The *rpoB* gene was first used in 1997 [44], and it provides better phylogenetic resolution than the 16S rRNA gene at both the species and subspecies levels [43]. Although it was not investigated further, likely due to the lower completeness of the *A. triatominarum* genome assembly (Figure 1, 137 of 440 BUSCO, 31.1% predicted as missing), its *rpoB* could not be identified, and therefore was excluded from phylogenetic analysis. Regardless, the resultant phylogenetic tree provides strong support for the bacterial species (with bootstrap values ≥ 97%), and groups primary symbionts into a distinct clade. The tree topology also reflects the relatedness of their insect hosts.

**Figure 3:**
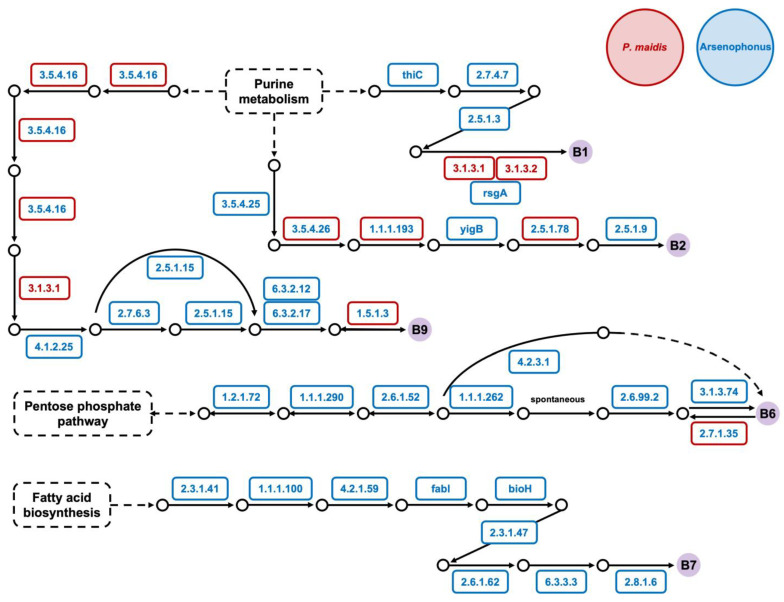
The biosynthetic pathway of B vitamins completed by *Arsenophonus* sp. and *P. maidis*. The KEGG pathway analysis was conducted, and enzyme commission numbers (EC numbers) are shown above. The EC numbers in red font within red boxes represent enzymes that were found in the *P. maidis* transcriptome; those in blue font within blue boxes represent enzymes that were found in the *Pm Arsenophonus* genome; and those in blue font within red boxes indicate enzymes that were found in both. B vitamins are shown in solid purple circles.

**Figure 4:**
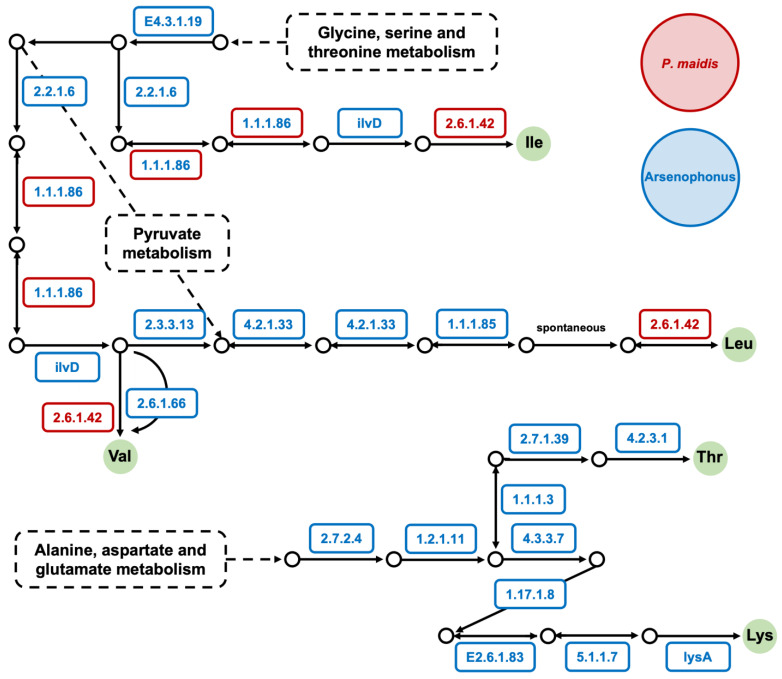
The biosynthetic pathway of essential amino acids completed by *Arsenophonus* sp. and *P. maidis*. The KEGG pathway analysis was conducted, and EC numbers are shown above. The EC numbers in red font within red boxes represent enzymes that were found in the *P. maidis* transcriptome; those in blue font within blue boxes represent enzymes that were found in the *Pm Arsenophonus* genome; and those in blue font within red boxes indicate enzymes that were found in both. Essential amino acids are shown in solid green circles.

To gain insights into the role *Pm Arsenophonus* sp. plays in *P. maidis,* we conducted KEGG pathway analysis on the *Arsenophonus* genome and previously reported *P. maidis* transcriptome [5]. Since some *Arsenophonus* spp. are reported to provide their insect hosts with B vitamins (Table 2) [10,15,16,45], we specifically interrogated components of the biosynthetic pathways for vitamins B1, B2, B5, B6, B7 and B9. Our KEGG pathway analysis suggests that *Pm Arsenophonus* sp. complements the biosynthetic pathways for vitamins B1, B2, B6, B7 and B9 in *P. maidis* (Figure 3). The nine enzymes for vitamin B7 production are encoded solely within *Pm Arsenophonus* sp., suggesting that the insect is dependent on the bacterium for B7 provisioning. We also examined components of biosynthetic pathways for essential amino acids, since symbionts in hemipterans are known for supplying essential amino acids to their insect hosts. KEGG analysis suggests that *Pm Arsenophonus* sp. provides components for the production of isoleucine, leucine, valine, threonine and lysine (Figure 4). The sole encoding of enzymes for threonine and lysine biosynthesis within the *Pm Arsenophonus* genome further emphasizes their symbiotic relationship. While such contributions of *Arsenophonus* spp. to essential amino acid provisioning in insect hosts have not been observed in previous studies (Table 2), our findings point to a potential novel aspect of the involvement of *Arsenophonus* sp. in the metabolism of *P. maidis*, meriting further investigation.

Whitten et al. [7] demonstrated the feasibility of symbiont-mediated RNAi in the kissing bug (*Rhodnius prolixus*) and the western flower thrips (*Frankliniella occidentalis*). They found that targeted insect genes were significantly knocked down by feeding the insects dsRNA-expressing symbiotic bacteria. Furthermore, the modified symbionts were able to successfully compete with the gut microbiota. While there is more to learn, the important role *Pm Arsenophonus* sp. plays in *P. maidis* and the availability of protocols for culturing and genetically modifying *Arsenophonus* spp. support *Pm Arsenophonus* sp. as an excellent candidate for use in symbiont-mediated RNAi.

## Figures and Tables

**Table 1:** Comparison between *Arsenophonus* genomes.

Name	*Arsenophonus nasoniae*	*Arsenophonus* sp.	*Arsenophonus lipopteni*	*Arsenophonus* *triatominarum*	
Accession No.	GCA_004768525.1	GCA_020268605.1	GCA_001534665.1	GCA_001640365.1	
Host	*Nasonia vitripennis*	*Apis mellifera*	*Lipoptena fortisetosa*	*Triatoma infestans*	
Sequencing	Oxford Nanopore MinION; PacBio RS II; Illumina MiSeq	Oxford Nanopore MinION; Illumina MiSeq	Illumina	PacBio	
Genome size (bp)	4,987,107	3,639,254	836,724	3,858,720	
GC content (%)	38.1 ^1^	37.7 ^1^	24.9 ^1^	38.3	
Pseudogene ^2^	1086	720	104	523	
Name	*Arsenophonus* sp. (ARAD)	*Arsenophonus* sp. (ARAF)	*Arsenophonus* sp. (ENCA)	*Arsenophonus* sp.	*Arsenophonus* sp. (this study)
Accession No.	GCA_900343015.1	GCA_900343025.1	GCA_002287155.1	GCA_000757905.1	
Host	*Aleurodicus disperses*	*Aleurodicus floccissimus*	*Entylia carinata*	*Nilaparvata lugens*	*Peregrinus maidis*
Sequencing	Illumina HiSeq 2000	Illumina HiSeq 2000	Illumina MiSeq	Illumina HiSeq 2000	PacBio
Genome size (bp)	663,125	3,001,875	3,228,533	2,953,863	4,888,380
GC content (%)	32.2 ^1^	37	39.5	37.6	41.3
Pseudogene ^2^	29	382	430	469	1123

^1^ Plasmid sequence was not included in the calculation of GC content. ^2^ The number of pseudogenes was roughly estimated by applying the criteria based on a previous study (E value < 10^−15^ and similarity < 80%) [32] to the predicted genes.

**Table 2:** Comparison among *Arsenophonus* spp.

Name	Host	Role and Function ^1^	Transmission	Reference
*Arsenophonus phytopathogenicus*	*Pentastiridius leporinus*	Plant pathogen of sugar beets vectored by *P. leporinus*	Maternal and horizontal (major) transmission by infecting the same sugar beet	[19]
*Arsenophonus nasoniae*	*Nasonia vitripennis* *Nasonia longicornis*	Reproductive parasite Killing sons, 80% of sons die	Maternal and horizontal transmission by infecting the same pupa host	[46]
*Arsenophonus* sp.	*Apis mellifera*	Providing B vitamins (B2, B6, B7, B9)	Horizontal transmission by social interactions (trophallaxis and/or general contact) and environmental acquisition	[18,45]
*Arsenophonus lipopteni*	*Lipoptena cervi*	Primary endosymbiont Providing B vitamins (B2, B6, B7)	Unclear	[15]
*Arsenophonus triatominarum*	triatomine bugs	Secondary endosymbiont No apparent effect on host fitness or reproduction	Vertical transmission (transovarially)	[17]
*Arsenophonus* sp. (ARAD)	*Aleurodicus disperses*	ARAD as primary endosymbiont ARAF and ARTV as secondary endosymbiont ARAD providing cofactors and B vitamins (B1, B2, B6, B7, B9)	Unclear	[16]
*Arsenophonus* sp. (ARAF)	*Aleurodicus floccissimus*			
*Arsenophonus* sp. (ARTV)	*Trialeurodes vaporariorum*			
*Arsenophonus* sp.	*Nilaparvata lugens*	Secondary endosymbiont Providing B vitamins	Maternal transmission	[10,47]

^1^ B vitamins in bold indicate that *Arsenophonus* sp. possesses all the genes in the biosynthetic pathway; B vitamins with an underline indicate that genes from both the *Arsenophonus* sp. and the host are required to complete the biosynthetic pathway; the remainder are not specified in references.

## Data Availability

The data are available at NCBI with the BioProject ID PRJNA1060725.
